# Machine learning prediction of nutritional status among pregnant women in Bangladesh: Evidence from Bangladesh demographic and health survey 2017–18

**DOI:** 10.1371/journal.pone.0304389

**Published:** 2024-05-31

**Authors:** Najma Begum, Mohd. Muzibur Rahman, Mohammad Omar Faruk

**Affiliations:** 1 Department of Statistics, Noakhali Science and Technology University, Noakhali, Bangladesh; 2 Department of Statistics, Jahangirnagar University, Savar, Dhaka, Bangladesh; Khulna University, BANGLADESH

## Abstract

**Aim:**

Malnutrition in pregnant women significantly affects both mother and child health. This research aims to identify the best machine learning (ML) techniques for predicting the nutritional status of pregnant women in Bangladesh and detect the most essential features based on the best-performed algorithm.

**Methods:**

This study used retrospective cross-sectional data from the Bangladeshi Demographic and Health Survey 2017–18. Different feature transformations and machine learning classifiers were applied to find the best transformation and classification model.

**Results:**

This investigation found that robust scaling outperformed all feature transformation methods. The result shows that the Random Forest algorithm with robust scaling outperforms all other machine learning algorithms with 74.75% accuracy, 57.91% kappa statistics, 73.36% precision, 73.08% recall, and 73.09% f1 score. In addition, the Random Forest algorithm had the highest precision (76.76%) and f1 score (71.71%) for predicting the underweight class, as well as an expected precision of 82.01% and f1 score of 83.78% for the overweight/obese class when compared to other algorithms with a robust scaling method. The respondent’s age, wealth index, region, husband’s education level, husband’s age, and occupation were crucial features for predicting the nutritional status of pregnant women in Bangladesh.

**Conclusion:**

The proposed classifier could help predict the expected outcome and reduce the burden of malnutrition among pregnant women in Bangladesh.

## Introduction

Nutritional status is the outcome of the biological phenomenon of food utilization and is a vital aspect of health. Good nutrition is linked to better health outcomes in infants, children, and mothers, more robust immune systems, reduced risk of non-communicable diseases (like diabetes and cardiovascular disease), and safer pregnancies and childbirths [1]. In contrast, malnutrition can lead to a range of issues, including low work productivity, higher chances of miscarriage, stillbirth, low birth weight, infant mortality, and fatal complications during pregnancy, delivery, and postpartum periods [2]. Malnutrition poses significant health risks. Globally, nearly 1.9 billion adults are either overweight or obese, and approximately 462 million adults are underweight [3]. Regarding nutrition, Bangladesh is experiencing a decline in underweight individuals but an upward trend in overweight and obese individuals [3]. Rural-urban disparities in unhealthy body mass index (BMI) categories are also a significant concern. According to the Bangladesh Demographic and Health Survey (BDHS) from 2017–2018, 13% of rural women are underweight, while 9% of urban women are underweight. In contrast, 43% of urban women are overweight or obese, compared to 28% of rural women who are obese [3, 4].

Approximately 200 million women become pregnant yearly, most residing in developing countries [5]. The nutrition of mothers during pregnancy is critical for the short- and long-term health of both the mother and her growing fetus [6]. A healthy pregnancy outcome is contingent upon good nutritional status before and during pregnancy. Maternal malnutrition poses significant health risks for both the pregnant mother and her children [7]. In Ethiopia, pregnant women’s undernutrition ranges from 21.8 to 43.1%. Rural women exhibit a higher prevalence of undernutrition [8, 9]. Malnutrition in pregnant mothers often goes unnoticed and unreported, resulting in insufficient attention given to the extent, consequences, and causes of this health issue [10]. Extensive research has been conducted on malnutrition’s impact on pregnant women’s health. Numerous factors contribute to malnutrition, including demographic, household, physical, socioeconomic, and cultural factors [11]. Previous studies have shown that individuals with a lower wealth index and less education are at a higher risk of being underweight, but the threat of being overweight is lower [12].

Machine learning is an intersection of artificial intelligence and statistical learning that explores large data sets to uncover unknown patterns or relationships [13]. Various studies have been conducted to identify the most informative risk factors and predict nutritional status using machine learning models, such as child malnutrition [14, 15] and malnutrition among women [2, 3], based on different demographic and health survey (DHS) datasets. Islam et al. (2022) utilized the Bangladesh Demographic Health and Survey (BDHS) 2014 dataset with 15,464 respondents and employed five different algorithms–NB, DT, SVM, ANN, and RF–to predict malnourished women. The RF classifier was found to have the highest accuracy (81.4%) and AUC (0.837) for underweight and accuracy (82.4%) and AUC (0.853) for overweight/obese [2]. Moreover, Mukuku et al. (2019) conducted a cross-sectional study with 263 children and employed an LR-based algorithm to predict nutritional status, revealing an AUC of 0.969, sensitivity of 93.5%, and specificity of 93.1% [16]. Another study by Hossain et al. (2022) applied six different machine learning algorithms to predict unintended pregnancies among married women in Bangladesh using the pregnancy intention of 1129 respondents. Among them, the elastic net regression (ENR) algorithm gained a higher AUC of 74.67% [17].

Researchers have recently used various machine-learning algorithms to study prediction performance [18]. All in all, machine learning is now being used everywhere in the research sector. Nowadays, machine learning is prevalent in health-related fields [13, 14, 19]. However, no research has considered machine learning algorithms to evaluate the nutritional status of pregnant women. The main objective of this study was to use various well-known machine learning algorithms to predict the nutritional status of pregnant women in Bangladesh and to identify the critical features of the best model with more accurate prediction.

## Methodology

### Data source and sampling design

The nutritional status of currently pregnant women data was extracted from the Bangladesh Demographic and Health Survey (BDHS), conducted in 2017–18, which is accessible online [4]. This study only included women currently pregnant and excluded all women who did not fall into the inclusion criteria. BDHS 2017–18 data comprise 20,127 ever-married women aged 15–49 who were interviewed. Among them, 18,895 were married women. However, only 1,129 currently pregnant women were included in this study. The purpose of BDHS was to collect household data to monitor and evaluate the health status of mothers and children, including nutrition, causes of death, newborn care, women’s empowerment, and more. United States Agency for International Development (USAID) provided financial assistance for this investigation in Bangladesh. Demographic Health Survey Authority employed a two-step stratified sampling procedure in the 2017–18 BDHS, where data was collected from eight divisions: Barisal, Chattogram, Dhaka, Khulna, Mymensingh, Rajshahi, Rangpur, and Sylhet. The survey used a list of enumerated areas (EAs) from Bangladesh’s population and housing census 2011 provided by the Bangladesh Statistics Office (BBS). In the 1^st^ sampling stage, 675 Eas were selected, of which 425 were from rural areas and 250 were from urban areas, with a probability proportional to the EA scale. In the second sampling stage, a complete household listing procedure was carried out in all selected Eas to provide a sampling frame for the systematic selection of 30 households per EA. This allowed for statistically accurate estimates of key demographic and health variables for the nation, rural, and urban areas separately [4].

### Study variables and measurement

#### Dependent variable

The study primarily focused on assessing the nutritional status of pregnant women by utilizing the body mass index (BMI) as a measure. According to the World Health Organization (WHO), BMI was categorized as underweight (BMI<18.5 kg/m2), normal weight (18.5≤BMI≤24.9 kg/m2), overweight (25.0≤ BMI<30.0 kg/m2), and obese (BMI≥30.0 kg/m2) [20]. However, for this study, overweight and obese women were classified as a single category.

This study tried to recommend the nutritional status of pregnant women. To begin with, we consider the current weight of the pregnant women. During normal pregnancy, women gain weight 11.5–16 kg, and it is essential to note that usual weight gain (UWG) is not affected by the height of the pregnant women [21, 22]. To calculate the pre-pregnancy weight of pregnant women, we have deduced the usual weight gain (UWG) during pregnancy from their current weight.

In the first trimester (1–13 weeks), the UWG is 0.5–2 kg and 0.35–0.5 kg/week for the second and third trimesters [21–25]. UWG for the first trimester is 0.5kg, with little weight gain experienced during the first trimester [21, 26]. In the second trimester, we divided it into two equal parts: the first half (14–20 weeks) and the second half (21–27 weeks). It is mentioned that the weight gain is typically lower during the first half of the second trimester [27]. Therefore, we consider UWG for this part to be 0.35 kg/week. It is also mentioned that the weight gain in the second half of the second trimester is comparatively higher than in the first half [23]. So, we consider the UWG 0.5kg/week for this part. In the third trimester, UWG is also 0.5kg/week [28].

So, at a glance, UWG during the first trimester or first three months is 0.5kg, and up to 20 weeks of gestation or during the first five months (0.5+0.35*7), it’s approximately 3 kg. For the subsequent months, it’s considered 2 kg per month. Using this information, we can calculate the pre-pregnancy weight of the pregnant women = current weight—UWG during pregnancy.

For example, if a pregnant woman is seven months into her pregnancy and her current weight is 80kg, and according to our discussion, UWG of 7kg, her pre-pregnancy weight would be 73kg. BIM calculated for this pregnant woman is = (73kg/ height in meters squared). Calculate the BMI for each respondent in the study using the formula (pre-pregnancy weight in kg/height in meters squared), which categorizes as underweight (<18.5 kg/m2), normal weight (18.5≤BMI≤24.9 kg/m2), and overweight (≥25.0 kg/m2). Previous literature conducted in Asian countries has used the BMI categories recommended by the World Health Organization (WHO) [2, 29–33]. Following this literature, we have used the WHO-recommended BMI categories in our analysis.

#### Independent variable

Table 1 presents the predictor names, types, descriptions, and categorizations based on previous relevant works [2, 3, 14, 15] The predictors included the respondent’s age, place of residence, region, religion, educational attainments, current employment status, wealth index, total number of children, number of living children, current pregnancy wanted, currently breastfeeding, access to mass media, age, occupation, and educational attainment of the partner, toilet facility, and sources of drinking water.

**Table 1:** Descriptions of the predictors along with their categorizations.

Serial	Variables	Variable type	Descriptions	Categorization
1	Respondent’s current age	Categorical	respondent’s age in years	15–19, 20–24, 25–29, 30–34, 35–49
2	Region	Categorical	The administrative region where respondents reside	Barisal, Chittagong, Dhaka, Khulna, Rajshahi, Rangpur, Sylhet
3	Residence	Categorical	Type of place of residence	Urban and Rural.
4	Highest educational level	Categorical	Respondent’s education level	no education, primary, secondary, higher
5	Religion	Categorical	Religion	Muslim, non-Muslim
6	Wealth index combined	Categorical	Respondents’ wealth index status	Poorest, poorer, middle, richer, richest
7	Total children ever born	Categorical	children ever born	0, 1–2, 3–4, ≥ 5
8	Number of living children	Categorical	living children	0, 1–2, ≥3
9	Current pregnancy wanted	Categorical	pregnancy status	no, yes
10	Currently breastfeeding	Categorical	The respondent is currently breastfeeding	no, yes
11	Husband/partner’s education level	Categorical	husband’s education level	no education, primary, secondary, higher
12	Respondent currently working	Categorical	Currently working status	no, yes
13	Husband/partner’s age	Categorical	husband’s age in years	<30, 31–49, >50
14	Access to mass media	Categorical	merging three variables: watching TV, reading the newspaper, and listening to the Radio.	no, yes
15	Husband/partner’s occupation	Categorical	Husband’s working status	farmer, daily laborer, employee, businessman
16	Toilet facility	Categorical	Type of toilet facility	hygienic, nonhygienic
17	Drinking water	Categorical	Sources of drinking water	improved, non-improved

### Data pre-processing

The BDHS 2017–18 data has been used for this study. First, reviewing the literature, we made a list of variables and extracted the selected variables from the BDHS data. Most of the features considered in this study were categorical, and a few were numeric. The numeric variable was also converted into categorical for the convenience of the study. Before model training, an extensive exploratory analysis was conducted. The categorical features of the dataset were encoded for numerical values. First, all variables’ frequency was calculated to check anomalies such as inconsistent values, missing observations, and outliers. The conflicting values were removed or replaced with consistent values. The missing values and outliers were deleted from the dataset.

#### Feature selection

We proceeded with variable or feature selection after removing any missing values. Variable selection aims to reduce data dimensions to minimize processing time and computation costs [34]. To enhance the overall predictive performance of the classification, we chose a subset of variables that significantly contributed to the target class. Identifying these by performing the chi-square (χ2) test between nutritional status (BMI) with each of the variables primary, which was adjusted for the complex survey design using second-order Rao–Scott corrections [35, 36]. And included those with a p-value < 0.05. Thirteen features met these criteria and were selected for developing the classification model. These features included the respondent’s age, region, place of residence, highest educational level, wealth index, total children ever born, number of living children, current pregnancy wanted, access to mass media, husband’s age, husband’s education level, husband’s occupation, and toilet facility. The S1 Table shows the features list from the chi-square test results, adjusted using second-order Rao–Scott corrections.

#### Dealing with imbalanced datasets

In this study on the BMI data of pregnant women, we noted a class imbalance, which could result in inaccurate or biased estimates of measures such as accuracy and precision. The percentage of overweight/obese pregnant women in Bangladesh was 15%, which may create an imbalanced distribution of the underlying classes and lead to biased and unreliable results while using ML. To overcome this issue, an oversampling approach named the Synthetic Minority Oversampling Technique (SMOTE) was implemented. This technique was developed by Nitesh Chawla [37].

#### Model validation

For ML approaches, the dataset is randomly divided into two distinct datasets: a training dataset that comprises 70% of the data and a test dataset that predicts the response variable and checks whether the expected outcome is similar to the actual outcomes, which include 30% of the primary dataset. All models were trained based on 10-fold cross-validation, designed to assess performance and optimize prediction models using ML techniques. The Statistical Package for Social Science (SPSS) 26 version and Python version 3.9.13 were used for data management and analysis.

### Feature transformation (FT)

Four feature selection techniques were applied to decrease the datasets’ spread equality, skewness, and linear and additive relationships (see details in Table 2). From these transformations, we evaluated the best one for which the best ML model can be extracted. The transformations we applied are Standardization, Min-Max Scaling, Log Scaling, and Robus Scaling. A brief description of the transformation has been presented in Table 2.

**Table 2:** Description of different FT methods.

FTs	Details	Formula
Standardization (FT1)	It converts different features into the range -1 to +1 [38].	$X_{scaled}=\frac{X_{i}-X_{mean}}{\sigma }$
Min-Max Scaling (FT2)	Min-max is a scaling technique where values are rescaled and shifted to a range between 0 and 1 [38].	$X_{scaled}=\frac{X_{i}-X_{min}}{X_{max}-X_{min}}$
Log Scaling (FT3)	It helps convert a skewed distribution to a normal distribution/less-skewed distribution [39].	*X*′ = log(*X*)
Robust Scaling (FT4)	The scaled values will have their median, and IQR set to 0 and 1, respectively [40].	$X_{scaled}=\frac{X_{i}-X_{median}}{IQR}$

### Machine learning algorithms

This research utilized ten machine learning algorithms to predict the nutritional status (underweight, normal weight, overweight/obese) of pregnant women in Bangladesh. The performance of these algorithms was evaluated based on model evaluation parameters. The ML algorithms used in this investigation include logistic regression (LR), decision trees (DT), random forest (RF), k-nearest neighbors (k-NN), support vector machine (SVM), Naïve Bayes (NB), adaptive boosting (ADB), extreme gradient boosting (XGB), gradient boost, and bagging were included in this analysis. A brief description of ML algorithms used in this study is provided in supplement A in the S1 File.

### Performance evaluation

Research supports using a variety of measures to assess and summarize a model’s performance, as no single measure can fully capture all aspects of a model. Methods such as accuracy, f1 score, precision, recall (sensitivity), and the area under the receiver operating characteristic curve should be employed to evaluate a model. Supplement B in the S1 File will discuss each performance evaluation parameter.

### Feature importance

Identifying important features is crucial to machine learning prediction. Feature importance rates illustrate the significance of each feature for decision-making purposes. We have utilized two distinct feature importance methods, namely (a) Mean Decrease Impurity (MDI) and (b) Permutation Importance (PI), to identify the significant features from the datasets. After analyzing these datasets, we determined the algorithm that yielded the best results.

### Ethical approval

BDHS 2017–2018 provided the publicly available secondary data for this study, which was conducted with ethical approval from the Institutional Review Boards of ICF Macro in Calverton, MD, USA, and Bangladesh Medical Research Council. All participants were informed of the study’s purpose, risks and benefits, future use of data, confidentiality, and anonymity, and they provided informed consent. We removed all identifier information before downloading the data from the BDHS website [4].

## Results

### Baseline characteristics

S2 Table depicts the background characteristics of the pregnant women participating in this study. The most significant percentage of respondents are from the Chittagong and Dhaka divisions (15.4%) and (15.3%), respectively. The highest proportion of mothers belongs to the 20–24 age group, accounting for 34.4%, while most of the respondents’ husbands aged between 20–30 years (77%), but there were still some pregnant women who were less than 20 years old and over 35 years. Besides, (48.5%) of pregnant women are in secondary education, and only (4.3%) of the respondents could not read and write. The education level of the partners of the respondents is distributed as follows: 12.6% have no education, 33.2% have primary education, 35.3% have secondary education, and 19% have higher education. The majority of pregnant women (67.2%) are not currently working, and most of the respondents’ husbands (35.4%) work as employees. Large numbers of pregnant women (50.5%) had 1–2 children. Most participants come from poor and rich wealth statuses (approximately 20% each), with only 18.8% belonging to middle-class families. Most pregnant women (64.7%) were involved in mass media, and 35.3% were not. 75% of women’s pregnancies had Intended, and only 6% were breastfeeding. Most of the respondents were from rural areas (64.6%) and had improved drinking water sources at home.

### Machine learning algorithm specifications

This study used specific machine learning algorithms, summarized in Table 3. To help prevent errors, 10-fold cross-validation was used to determine the best parameters for these algorithms.

**Table 3:** Specifications of machine learning algorithms.

Algorithm	Specifications
Logistic Regression (LR)	C: 1.0, penalty: 12
Decision Tree (DT)	criterion: entropy, max depth: None, min samples leaf: 1, min samples split: 2
Random Forest (RF)	criterion: Gini, max depth: 9, max features: log2, max-leaf nodes: 9, n estimators: 150
K-nearest neighbor (KNN)	n neighbors = 1
Support Vector Machine (SVM)	C: 10, gamma: 1, kernel: rbf
Naïve Bayes (NB)	Probability distribution: Gaussian
Adaptive Boosting (ADB)	Learning rate: 0.01, n estimators: 100
eXtreme Gradient Boosting (XGB)	Learning rate: 0.2, max depth: 5, min child weight:1, n estimators: 300
Gradient boosting (GB)	Learning rate: 0.5, max depth: 7, n estimators: 50}
Bagging	max features: 0.7, max samples: 1.0, n estimators: 100}

### Machine learning algorithms performance evaluation

This study applied four different feature transformation (FT) methods—Standardization, Min-Max, log, and Robust (referred to as FT1, FT2, FT3, and FT4)—along with ten machine learning (ML) algorithms to classify the nutritional status of pregnant women. The algorithms were evaluated based on various performance parameters, including accuracy, kappa statistics, precision, recall, f1 score, and AUC value. Tables 4–8 present each algorithm’s classification accuracy, kappa statistics, precision, f1 score, and recall. Tables 9–12 also show prediction results for underweight and overweight/obese classes, including AUC, precision, f1 score, and recall. The study also evaluated the performance of these ML algorithms without any transformation techniques, and the results showed that using FT methods improved the accuracy of the classification and other performance parameters. These results are reported in S3 Table.

**Table 4:** Accuracy (%) of different algorithms for overall nutritional status.

FT Methods	DT	RF	KNN	NB	LR	SVM	ADB	GB	XGB	Bagging
FT1	63.12	74.45	68.66	50.33	53.93	54.28	61.95	68.93	72.46	69.36
FT2	64.27	73.69	66.90	52.59	54.35	54.65	62.81	69.46	72.02	68.03
FT3	64.90	74.55	67.47	51.84	55.41	55.27	61.68	69.46	72.31	69.89
**FT4**	66.18	**74.75**	67.71	50.25	54.60	54.72	61.23	68.89	72.01	70.45

The accuracy of the pregnant women’s nutritional status is shown in Table 4, and revealed the RF with FT4 gained the highest accuracy (74.75%) compared to other classifiers.

**Table 5:** Kappa statistics (%) of different algorithms for overall nutritional status.

FT Methods	DT	RF	KNN	NB	LR	SVM	ADB	GB	XGB	Bagging
FT1	45.09	57.09	43.80	28.34	29.67	30.38	29.67	53.54	55.84	53.00
FT2	45.68	55.36	48.87	51.31	32.14	31.44	32.14	52.50	54.60	51.80
FT3	43.32	57.88	43.18	28.37	30.91	32.90	30.91	51.08	55.58	50.49
**FT4**	40.54	**57.91**	44.34	22.57	32.75	31.25	32.75	53.55	55.08	52.98

Table 5 shows that RF had the highest Kappa score (57.91%) with FT4.

**Table 6:** Precision (%) of different algorithms for overall nutritional status.

FT Methods	DT	RF	KNN	NB	LR	SVM	ADB	GB	XGB	Bagging
FT1	63.03	71.04	61.38	50.28	50.99	50.32	59.46	69.20	71.14	68.73
FT2	63.40	70.57	64.88	51.78	53.08	51.29	58.83	68.82	70.05	67.52
FT3	61.85	72.37	60.57	50.28	51.76	53.09	58.53	67.67	70.97	67.15
**FT4**	60.12	**73.36**	60.83	46.37	53.64	51.48	60.33	69.62	70.73	69.08

Table 6 reveals the precision performance of nutritional status for pregnant women. The RF algorithm performed the best, at 73.36%, with the FT4 method.

**Table 7:** Recall (%) of different algorithms for overall nutritional status.

FT Methods	DT	RF	KNN	NB	LR	SVM	ADB	GB	XGB	Bagging
FT1	63.38	70.90	62.54	52.34	53.18	53.68	60.20	69.06	70.57	68.73
FT2	63.71	70.23	65.89	54.01	54.68	54.18	59.53	68.23	69.73	67.89
FT3	62.21	71.91	62.21	52.17	53.85	55.18	59.20	67.39	70.40	67.06
**FT4**	60.37	**73.08**	62.88	48.33	55.18	54.18	60.87	69.06	70.07	68.73

From Table 7, it was found that the RF algorithm with the FT4 had the highest recall score at 73.08%.

**Table 8:** f1 score (%) of different algorithms for overall nutritional status.

FT Methods	DT	RF	KNN	NB	LR	SVM	ADB	GB	XGB	Bagging
FT1	63.02	70.85	60.20	50.18	51.50	49.22	59.29	69.07	70.65	68.72
FT2	63.20	70.22	63.27	51.31	53.22	49.78	58.67	67.85	69.71	67.62
FT3	61.93	71.90	59.99	49.65	51.95	50.65	58.45	67.36	70.44	67.08
**FT4**	60.17	**73.09**	59.50	46.53	53.88	50.52	59.89	69.19	70.18	68.87

In the study, Table 8 displayed the f1 score for ten algorithms, and the RF algorithm paired with the FT4 method had the highest f1 score, at 73.09%. The FT4 method for the RF algorithm yielded the best results among all the algorithms tested. Thus, for this study, robust transformation is applied to the data, and then the performance of the different classifiers is evaluated based on this transformation.

**Table 9:** AUC (%) of different algorithms for the underweight and overweight/obese.

Nutritional Status	FT Methods	DT	RF	KNN	NB	LR	SVM	ADB	GB	XGB	Bagging
Underweight	FT1	59	66	51	62	55	54	68	73	72	73
	FT2	62	70	47	64	47	47	74	**78**	**78**	**78**
	FT3	64	68	47	64	47	47	65	75	73	72
	FT4	58	66	50	64	51	50	68	75	72	73
Overweight/Obese	FT1	64	79	52	70	61	60	74	84	82	79
	FT2	66	75	48	71	52	52	77	84	83	83
	FT3	63	73	49	72	52	52	77	**85**	83	83
	FT4	66	79	51	71	57	56	76	82	82	81

**Fig 1:**
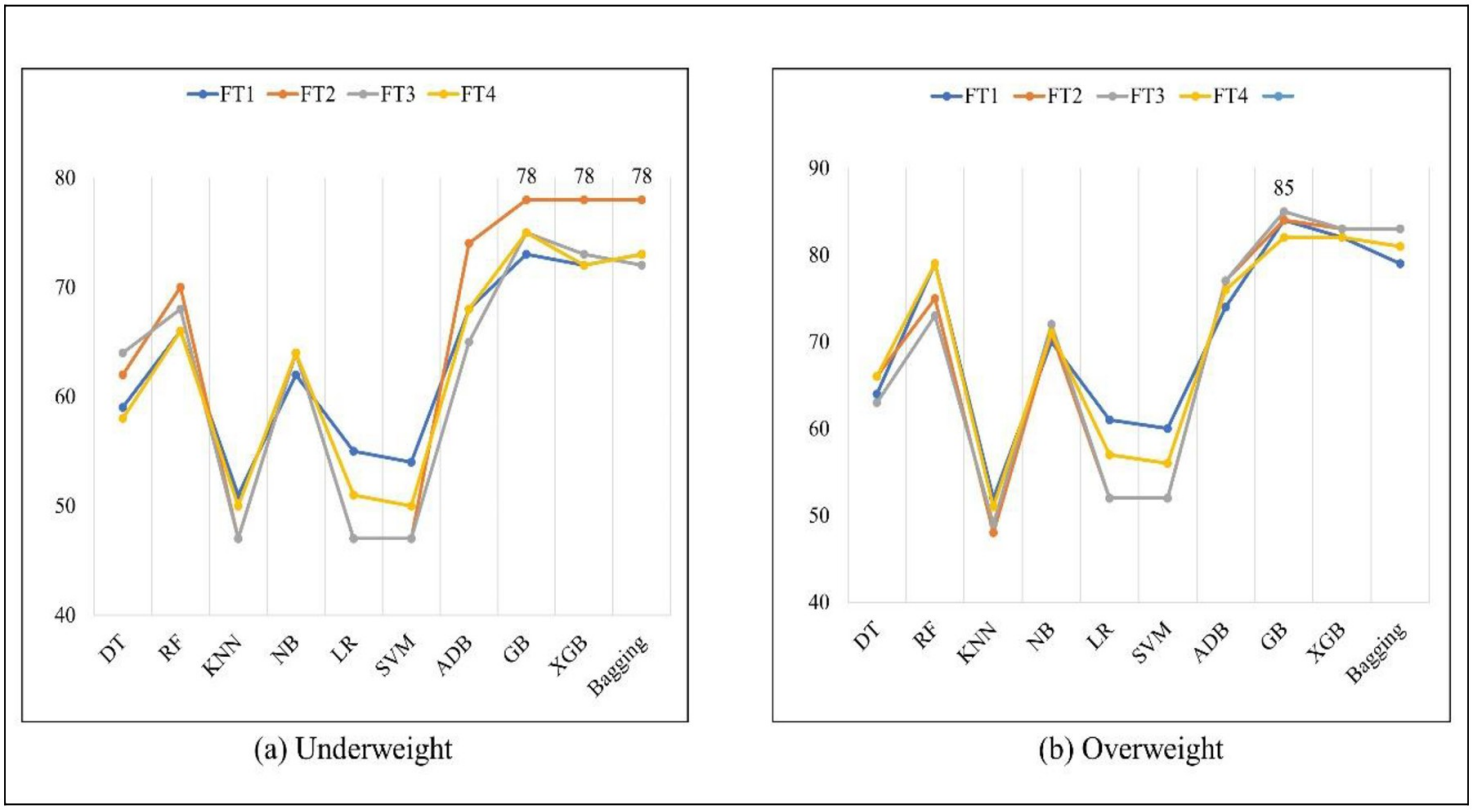
AUC (%) of different algorithms for underweight and overweight/obese classes.

Table 9 presents the AUC value predictions for the underweight and overweight/obese categories. The GB algorithm achieved an AUC value of 85.0% using the FT3 method for the overweight/obese class. Fig 1 exposes the highest AUC value for both categories across different algorithms.

**Table 10:** Precision (%) of different algorithms for the underweight and overweight/obese.

Nutritional Status	FT Methods	DT	RF	KNN	NB	LR	SVM	ADB	GB	XGB	Bagging
Underweight	FT1	66.02	74.74	61.69	54.83	58.09	56.27	60.44	71.14	74.47	69.52
	FT2	67.86	73.94	66.53	56.22	56.94	55.65	60.87	73.62	72.34	68.72
	FT3	61.00	**76.76**	61.48	54.62	56.62	57.42	59.62	69.84	73.66	65.70
	FT4	60.00	**76.17**	62.40	51.30	58.70	57.09	59.91	73.58	74.47	70.53
Overweight/Obese	FT1	66.82	77.95	66.11	55.61	57.99	54.88	63.96	74.19	79.46	75.68
	FT2	66.37	78.12	67.47	57.03	60.17	56.20	63.04	74.29	78.42	74.50
	FT3	70.65	78.76	67.86	54.15	59.26	55.31	63.23	73.54	78.80	78.57
	FT4	68.69	**82.01**	66.93	52.30	59.91	55.38	64.13	75.69	78.69	79.66

**Fig 2:**
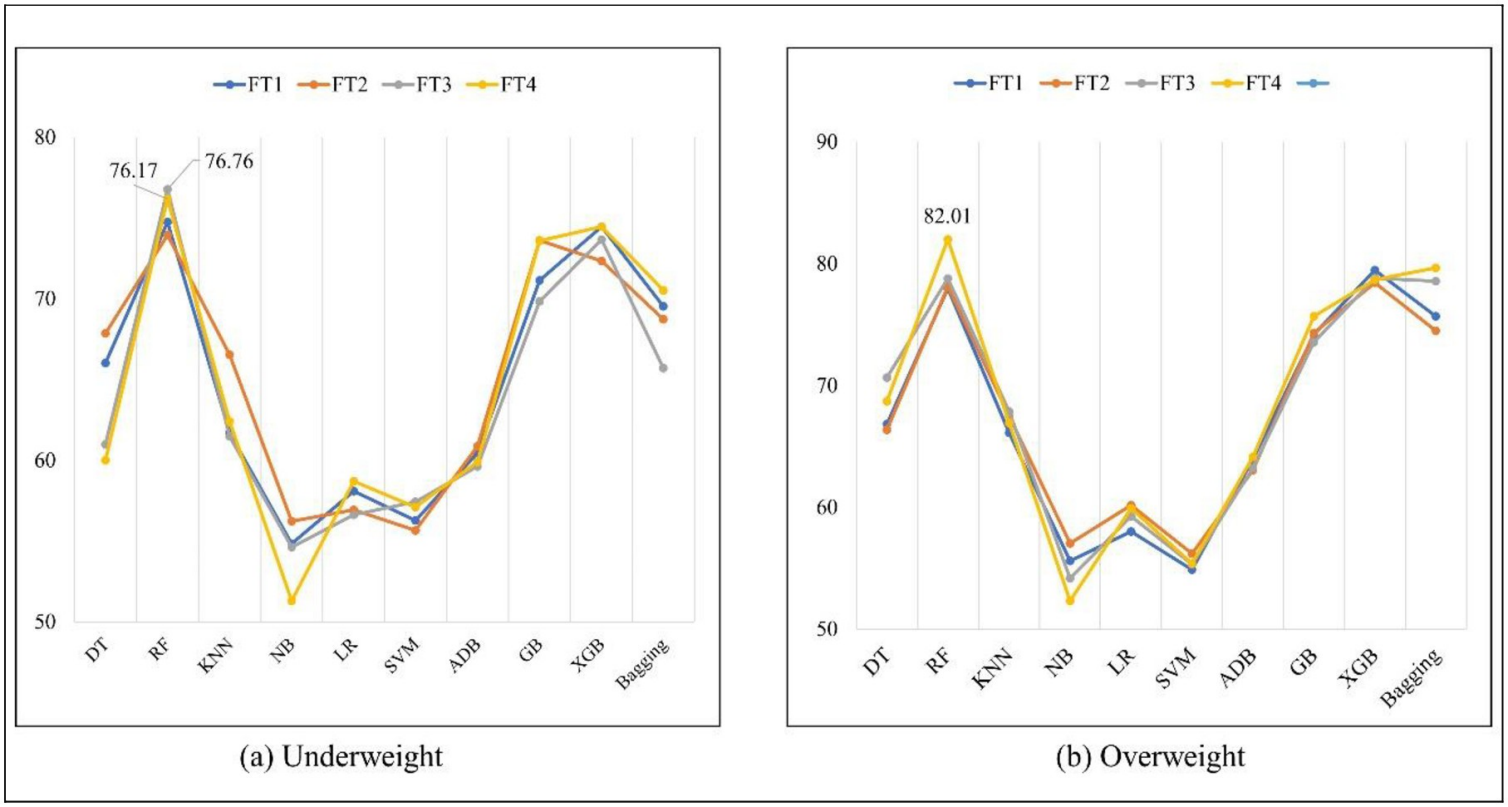
Precision (%) of different algorithms for underweight and overweight/obese.

Table 10 shows the precision outcome. The RF algorithm achieved the highest result of 76.76% with the FT3 method; the second highest precision of 76.17% was achieved by the FT4 method for the underweight class and 82.01% for the overweight/obese class. Fig 2 demonstrates the highest precision result for different algorithms for these classes.

**Table 11:** f1 score (%) of different algorithms for the underweight and overweight/obese.

Nutritional Status	FT Methods	DT	RF	KNN	NB	LR	SVM	ADB	GB	XGB	Bagging
Underweight	FT1	64.30	70.56	65.81	59.66	61.14	63.31	61.54	68.42	69.14	68.38
	FT2	64.41	68.64	70.15	58.22	56.81	59.35	59.43	63.16	67.16	67.76
	FT3	58.51	70.65	66.67	57.14	56.88	62.16	59.07	65.02	67.99	64.15
	FT4	57.55	**71.71**	66.81	52.80	60.40	62.34	61.92	69.27	69.14	68.87
Overweight/Obese	FT1	71.94	80.85	75.24	61.39	63.50	63.23	70.47	75.20	80.33	76.50
	FT2	73.27	80.43	78.14	66.82	68.11	67.69	70.56	79.80	80.32	78.22
	FT3	74.35	81.28	75.06	63.13	67.92	66.52	69.80	75.14	79.45	78.79
	FT4	71.77	**83.78**	77.78	59.52	66.00	64.35	70.79	75.69	79.12	78.77

**Fig 3:**
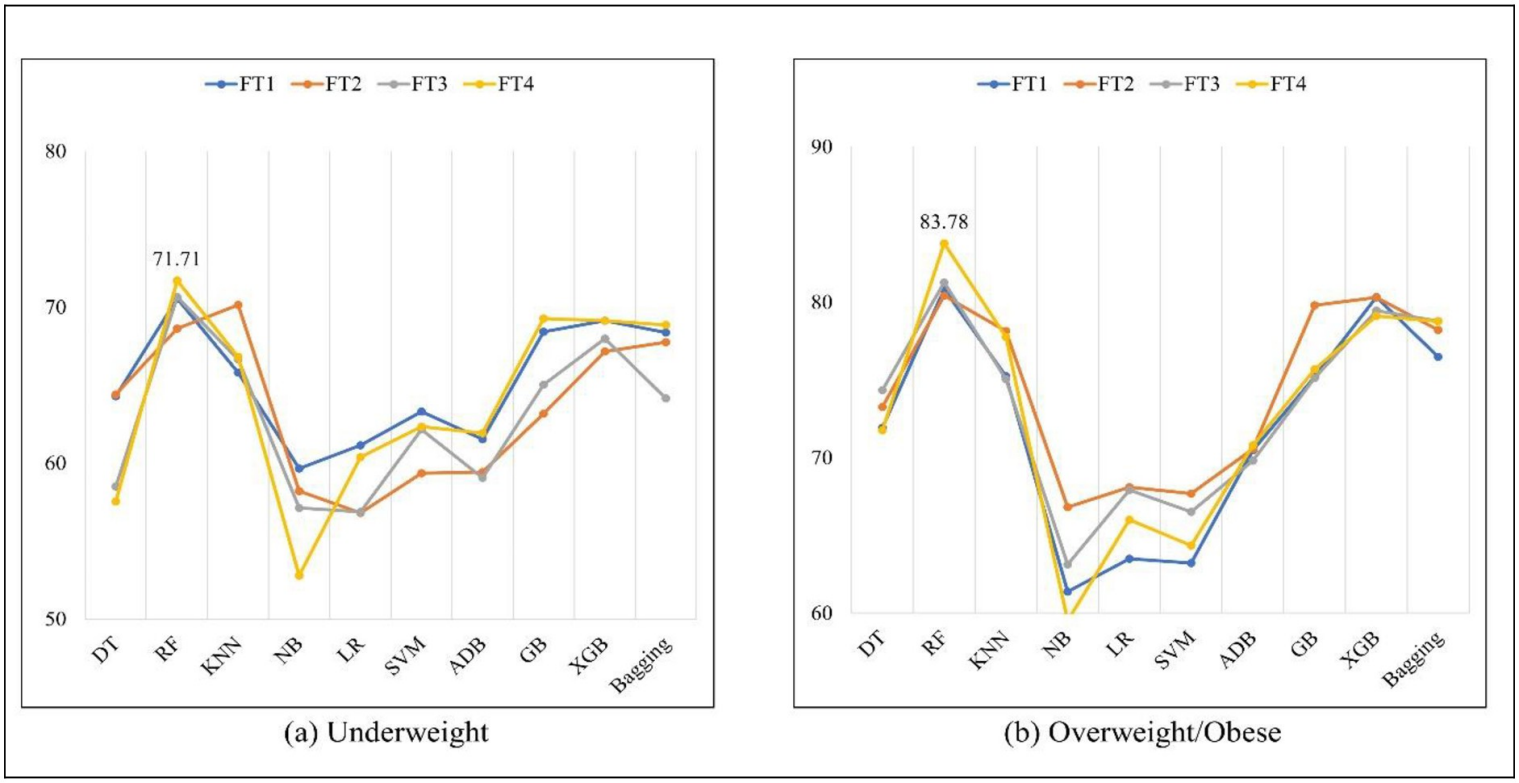
f1 score (%) of different algorithms for underweight and overweight/obese.

The f1 score performance was presented in Table 11, and it was found that the FT4 method under the RF algorithm attained the maximum f1 score of 71.71% for the underweight class and 83.78% for the overweight/obese class. Fig 3 shows the best f1 score results for different algorithms for both classes.

**Table 12:** Recall (%) of different algorithms for the underweight and overweight/obese.

Nutritional Status	FT Methods	DT	RF	KNN	NB	LR	SVM	ADB	GB	XGB	Bagging
Underweight	FT1	62.67	66.82	70.51	65.44	64.52	72.35	62.67	65.90	64.52	67.28
	FT2	61.29	64.06	**74.19**	60.37	56.68	63.59	58.06	55.30	62.67	66.82
	FT3	56.22	65.44	72.81	59.91	57.14	67.74	58.53	60.83	63.13	62.67
	FT4	55.30	67.74	71.89	54.38	62.21	68.66	64.06	65.44	64.52	67.28
Overweight/Obese	FT1	77.90	83.98	87.29	68.51	70.17	74.59	78.45	76.24	81.22	77.35
	FT2	81.77	82.87	**92.82**	80.66	78.45	85.08	80.11	86.19	82.32	82.32
	FT3	78.45	83.98	83.98	75.69	79.56	83.43	77.90	76.80	80.11	79.01
	FT4	75.14	85.64	**92.82**	69.06	73.48	76.80	79.01	75.69	79.56	77.90

**Fig 4:**
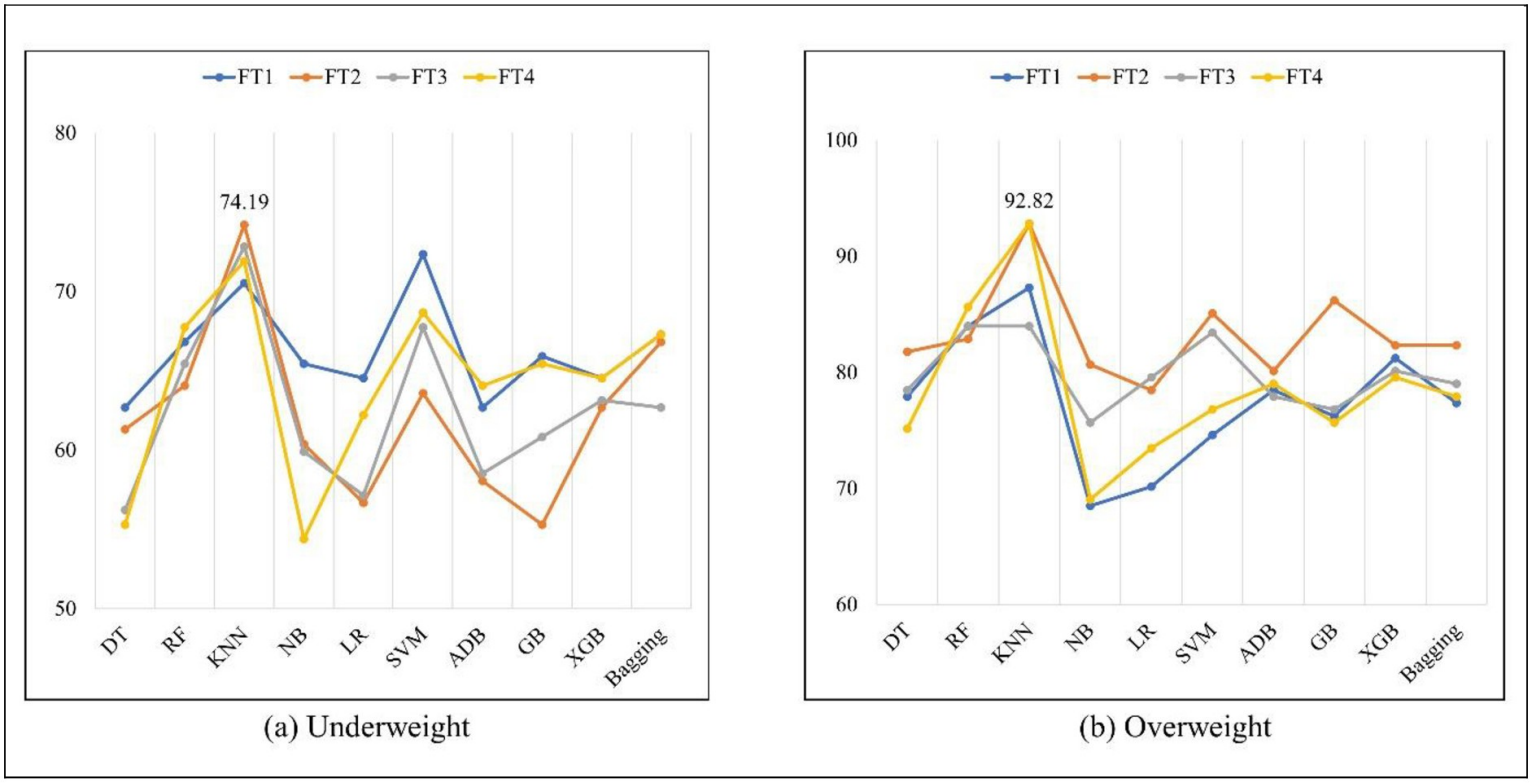
Recall (%) of different algorithms for underweight and overweight/obese.

Finally, Table 12 revealed that the KNN algorithm and FT2 and FT4 feature transformation methods yielded 74.19% and 92.82%, respectively. Fig 4 shows the highest recall value for different algorithms for both classes.

### Variable importance from best performing algorithm

**Fig 5:**
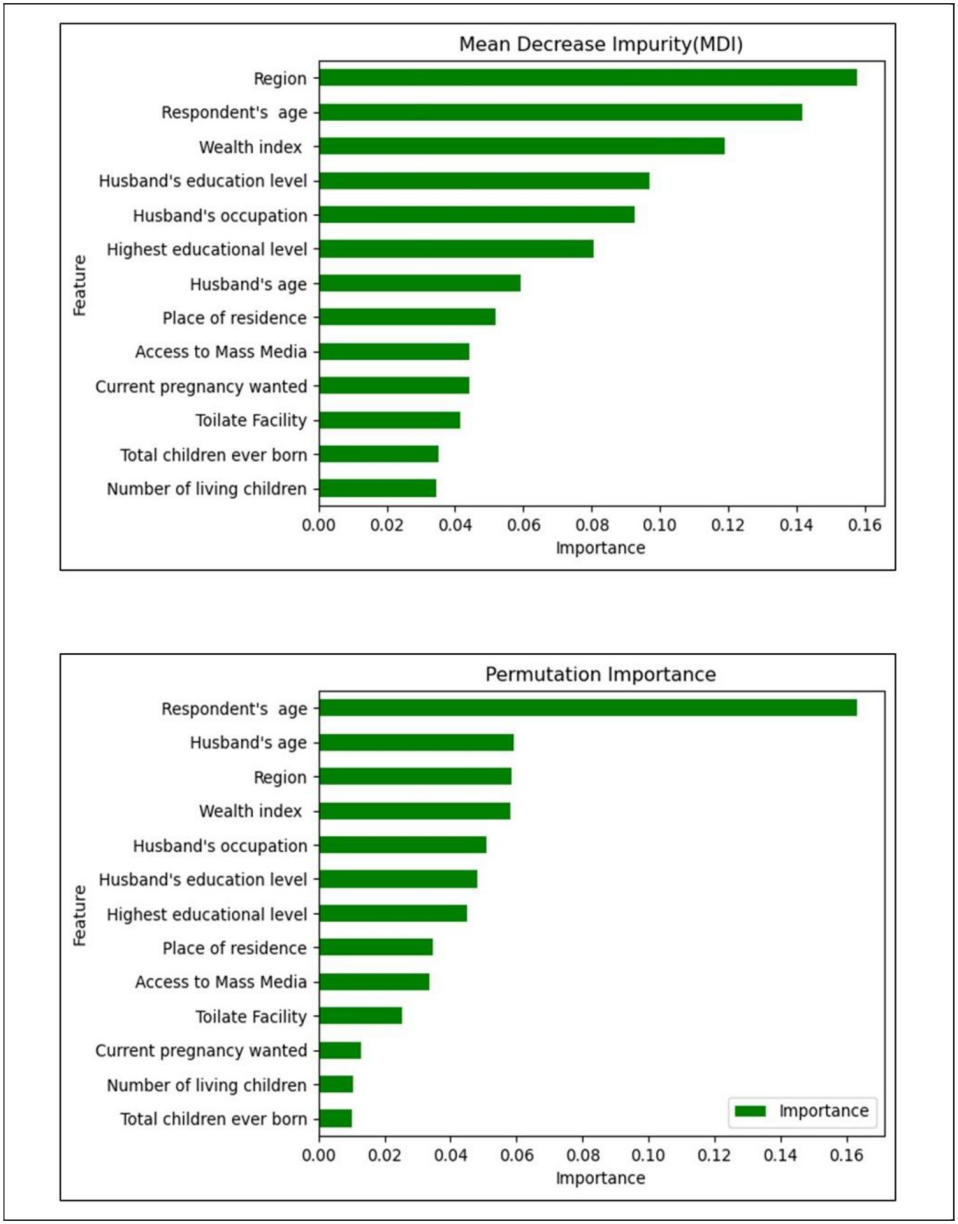
Variable importance from the best model (Random Forest).

After evaluating machine learning, two different feature importance approaches, such as MDI and PI, were implemented for the RF algorithm with robust scaling to utilize and rank the significant features of the datasets. The factors, including respondent’s current age, wealth index, region, husband’s education level, husband’s age, and occupation, were the most important features of the nutritional status of pregnant women. In contrast, variables such as total number of children ever born, religion, number of living children, and toilet facility were found to be the least predictive based on the all-features importance methods (Fig 5). S4 Table represents the important features rank of robust transformed datasets for the RF algorithm.

## Discussion

To date, many prediction models can identify the nutritional status of children and women in Bangladesh [2, 3, 13]. However, there is a lack of research on the potential use of machine learning techniques to predict the nutritional status of pregnant women in Bangladesh. The main aim of this study is to predict the nutritional status (underweight, overweight/obese) of currently pregnant women in Bangladesh. This study applied four different feature transformation (FT) methods and then ten well-known machine learning algorithms such as decision tree, logistic regression, random forest, support vector machine, k-nearest neighbor, naïve Bayes, adaptive boosting (ADB), eXtreme Gradient Boosting (XGB), gradient boost and bagging. All models were trained using 10-fold cross-validation on the training data set.

The results of this study revealed that the FT4 or robust transformation is the best in the case of pregnant women’s nutritional status as it achieved the highest performance parameter for all classifiers. RF algorithm gained the highest accuracy (74.75%), kappa statistics (57.91%), precision (73.36%), recall (73.08%), and f1 score (73.09%) among all algorithms applied in the investigation with FT4 or robust scaling. The RF classifier had a high precision of 76.76% and an f1 score of 71.71% for the underweight class, while for the overweight class, the precision was 82.01%, and the f1 score was 83.78%.

In a study conducted by Balabaeva et al. [41], the impact of various feature scaling methods on heart failure patient datasets was examined and used LR, XGB, DT, and RF algorithms with scaling methods such as Standard Scaler, Max Abs Scaler, MinMax Scaler, Quantile Transformer, and Robust scaler. The study found that RF demonstrated better performance with Standard Scaler and Robust Scaler, which is consistent with our findings. A study conducted by M. Ahsan on a dataset with heart disease patients to evaluate eleven machine learning (ML) algorithms and six different data scaling methods such as Normalization, MinMax, Standscale, MaxAbs, Quantile Transformer, and Robust Scaler and gained that CART algorithm, along with Quantile Transformer, or Robust Scaler, outperforms all other ML algorithms [40].

Islam et al. discovered that the RF algorithm has the best prediction accuracy and the highest AUC score compared to other machine learning algorithms for health issues, including women’s nutritional status [2]. Khudri et al. conducted a study that found the ADB, RF, and XGB algorithms were the most effective at predicting women of childbearing age’s nutritional status [3], supporting this study’s findings. J. Ali et al. [42] developed a nutritional prediction model for Pakistani women using a Support Vector Machine, Logistic Regression, Random Forest, K-nearest neighbor, and Naïve Bayes algorithms. They found that Random Forest had the highest accuracy. B. Alamma et al. [43] used Random Forest (RF) and Decision Tree (DT) classifiers to analyze risk factors for obesity and overweight women in their research. They found that the Random Forest algorithm produced the best results with an accuracy and f1 score of 77% and 75%, respectively. Dunstan conducted a study on predicting nationwide obesity from food sales and found that RF had the best performance, which supports the findings of this study [44]. Talukder and Ahammed applied RF, LR, SVM, k-NN, and LDA algorithms to predict malnutrition in under-five Bangladeshi children. They found that the RF algorithm performed the best, with a specificity of 69.76%, sensitivity of 94.66%, and accuracy of 68.51% [14]. Another study to predict under-five malnutrition in Bangladesh, conducted by S. Ahmed et al., showed the best performance by the RF algorithm with an accuracy of 70.1% and 72.4% and AUC of 69.8% and 70% for stunting and underweight, respectively [45]. In a recent study on predicting childhood anemia in Bangladesh, Khan and colleagues showed that the RF algorithm achieved a height accuracy of 68.53% with a specificity of 66.41%, sensitivity of 70.73%, and AUC of 0.6857 [13]. Rahman et al. discovered that the RF algorithm achieved the highest AUC of 0.6590, accuracy of 0.8890, specificity of 0.9789, sensitivity of 0.0480, f1 score of 0.0771, and precision of 0.1960 for infant mortality in Bangladesh compared to other algorithms [46]. The Random Forest algorithm outperformed all other algorithms (total accuracy: 95%; area under ROC curve: 93%; Kappa Coefficient: 66%) in Ahmadi’s study on predicting low birth weight [47]. S. Rahman et al. [48] implemented three ML classifiers (support vector machine, LR, and random forest) to predict malnutrition in children. They achieved the maximum accuracy of 87.7% for wasted, 88.3% for stunted, and 85.7% for underweight, obtained by the RF algorithm. Random Forest performed better than other algorithms in Chilyabanyama’s research on predicting stunting among children under five in Zambia, which supports the current investigation’s findings [49].

In addition to identifying the best predictive models, this study also determined the essential features predicting nutritional status among currently pregnant women in Bangladesh based on the best algorithm found in this study. Based on the important feature score for RF, algorithms suggested that the wealth index, respondent age, region, husband education level, and husband’s age and occupation are the six most important features for predicting the nutritional status of pregnant women in Bangladesh. Household wealth status is a significant factor in determining maternal health care. As per the findings of this study, mothers with poor socioeconomic status face a greater risk of being underweight than those with high socioeconomic status, which is consistent with a previous study [50]. This research aligns with previous studies that have linked wealth index and working women to maternal underweight and overweight/obesity [51]. Respondent age is a vital indicator of the nutritional status of pregnant women. Some previous studies revealed that respondent age during the third trimester of pregnancy is a risk factor for developing malnutrition [52, 53]. The husband’s age is also an important feature in the nutritional status of pregnant women. A study found that being overweight is more prevalent among women whose husbands are aged 31 years or above (29%) [3]. The current study also revealed that pregnant women whose husband’s education level is a significant factor related to nutrition, which is consistent with the former studies done by M. Fite [54] and Hossain [32]. According to a study conducted in a rural area of Assam, India, it was observed that the incidence of malnutrition among pregnant women was significantly associated with the occupation of their husbands. The study reported a strong positive relationship between BMI and the husband’s occupation, which supports this study’s results [55]. Another study by M. Fite et al. showed that pregnant women’s nutritional status and dietary practices can significantly impact their husbands’ occupation [54]. According to a study of women living in Bangladesh, the location of residence is the most important factor in pregnant women’s health status. This study’s findings align with previous research conducted by M. Islam [2] and another nationally representative study, which used BDHS data [56].

Despite their usefulness, ML models may have limitations, such as not providing odds ratios or coefficients to indicate the direction of the relationship between important features. Knowing the direction of the association of each feature’s importance would improve the design and implementation of interventions to prevent malnutrition among pregnant women in Bangladesh.

## Strengths and limitations

It is important to note that the study has limitations as it relies on cross-sectional data, which restricts its ability to access supplementary information about other related factors. However, it has been suggested that by combining these factors, the predictive power and AUC of the algorithms could potentially increase. Another limitation is that the study’s analysis did not adjust for the sampling weight. Despite these limitations, the study’s strength lies in identifying the best ML algorithm using various performance evaluation techniques, which is a significant contribution to the field of research.

## Conclusions

Malnutrition is a significant concern for the health of developing nations. This paper aims to conduct a comprehensive study that compares and assesses the effectiveness of various machine learning (ML) algorithms in predicting the nutritional status of pregnant women in Bangladesh. To summarize, we applied FT methods to the datasets and utilized various algorithms to analyze the transformed data and evaluate their performance. The best performance was found in this study of the RF algorithm for a robust scaling method. According to the RF algorithm, the most important features that determine the nutritional status of pregnant women in Bangladesh are the respondent’s age, wealth index, region, husband’s education level, husband’s age, and occupation. This research will assist healthcare providers and policymakers develop a framework for implementing necessary interventions and care practices to prevent severe complications and reduce the burden of nutritional status concerns.

## Supporting information

S1 Table Association between pregnant women’s nutritional status (BMI) and demographic and socio-economic characteristics. (DOCX)

S2 Table Background characteristics of the pregnant women in Bangladesh. (DOCX)

S3 Table Evaluation of prediction performance (%) of different ML Algorithms for Overall nutritional status, underweight, and overweight/Obese without any FT methods. (DOCX)

S4 Table Feature importance ranking for the best-performing algorithm. (DOCX)

S1 File (DOCX)
