# Supplementary material for: Machine learning prediction of nutritional status among pregnant women in Bangladesh: Evidence from Bangladesh demographic and health survey 2017–18
Source: PLoS One. 2024 May 31;19(5):e0304389. doi: 10.1371/journal.pone.0304389 (PMC11142495; doi:10.1371/journal.pone.0304389)
Supplement: S1 Table — (DOCX) [file pone.0304389.s001.docx]

**S1 Table:** Association between nutritional status (BMI) of pregnant women with demographic and socio-economic characteristics

| **Variables** | **Underweight**  **n (%)** | **Normal weight n (%)** | **Overweight**  **n (%)** | †**P-value** |
| --- | --- | --- | --- | --- |
| **Respondent's age** |  |  |  |  |
| 15-19 | 125(39.8%) | 177(56.4%) | 12(3.8%) |  |
| 20-24 | 97(25.0%) | 236(60.8%) | 55(14.2%) |  |
| 25-29 | 45(17.6%) | 139(54.5%) | 71(27.8%) | <0.001 |
| 30-34 | 18(12.5%) | 91(63.2%) | 35(24.3%) |  |
| 35-49 | 3(10.7%) | 21(75.0%) | 4(14.3%) |  |
| **Region** |  |  |  |  |
| Barisal | 25(21.9%) | 69(60.5%) | 20(17.5%) |  |
| Chittagong | 47(27.0%) | 102(58.6%) | 25(14.4%) |  |
| Dhaka | 39(22.5%) | 96(55.5%) | 38(22.0%) | <0.001 |
| Khulna | 22(18.2%) | 76(62.8%) | 23(19.0%) |  |
| Mymensingh | 36(24.7%) | 94(64.4%) | 16(11.0%) |  |
| Rajshahi | 31(25.6%) | 75(62.0%) | 15(12.4%) |  |
| Rangpur | 30(23.8%) | 71(56.3%) | 25(19.8%) |  |
| Sylhet | 58(37.7%) | 81(52.6%) | 15(9.7%) |  |
| **Place of Residence** |  |  |  |  |
| Urban | 81(20.3%) | 226(56.5%) | 93(23.3%) | <0.001 |
| Rural | 207(28.4%) | 438(60.1%) | 84(11.5%) |  |
| **Religion** |  |  |  |  |
| Muslim | 265(25.6%) | 607(58.6%) | 163(15.7%) | 0.153 |
| Non-Muslim | 23(24.5%) | 57(60.6%) | 14(14.9%) |  |
| **Highest educational level** |  |  |  |  |
| No education | 13(27.1%) | 31(64.6%) | 4(8.3%) |  |
| Primary | 103(34.0%) | 173(57.1%) | 27(8.9%) |  |
| Secondary | 133(24.3%) | 327(59.7%) | 88(16.1%) | <0.001 |
| Higher | 39(17.0%) | 133(57.8%) | 58(25.2%) |  |
| **Wealth index** |  |  |  |  |
| Poorest | 86(37.2%) | 132(57.1%) | 13(5.6%) |  |
| Poorer | 65(28.6%) | 140(61.7%) | 22(9.7%) |  |
| Middle | 53(25.0%) | 128(60.4%) | 31(14.6%) | <0.001 |
| Richer | 51(22.0%) | 138(59.5%) | 43(18.5%) |  |
| Richest | 33(14.5%) | 126(55.5%) | 68(30.0%) |  |
| **Total children ever born** |  |  |  |  |
| 0 | 137(30.9%) | 254(57.2%) | 53(11.9%) |  |
| 1-2 | 125(22.2%) | 328(58.3%) | 110 (19.5%) | <0.001 |
| 3-4 | 21(20.2%) | 70(67.3%) | 13(12.5%) |  |
| 5+ | 5(27.8%) | 12(66.7%) | 1(5.6%) |  |
| **Number of living children** |  |  |  |  |
| 0 | 140(29.9%) | 272(58.0%) | 57(12.2%) |  |
| 1-2 | 127(22.3%) | 332(58.2%) | 111(19.5%) | <0.001 |
| 3+ | 21(23.3%) | 60(66.7%) | 9(10.0%) |  |
| **Current pregnancy wanted** |  |  |  |  |
| No | 77(27.3%) | 174(61.7%) | 31(11.0%) | <0.015 |
| Yes | 211(24.9%) | 490(57.9%) | 146(17.2%) |  |
| **Currently breastfeeding** |  |  |  |  |
| No | 264(24.9%) | 626(59.0%) | 171(16.1%) | 0.427 |
| Yes | 24(35.3%) | 38(55.9%) | 6(8.8%) |  |
| **Access to Mass Media** |  |  |  |  |
| No | 142(35.7%) | 218(54.8%) | 38(9.5%) | <0.001 |
| Yes | 146(20.0%) | 446(61.0%) | 139(19.0%) |  |
| **Respondent working status** |  |  |  |  |
| No | 192(25.3%) | 448(59.0%) | 119(15.7%) | 0.508 |
| Yes | 96(25.9%) | 216(58.4%) | 58(15.7%) |  |
| **Husband's age** |  |  |  |  |
| <30 | 201(31.6%) | 369(58.0%) | 66(10.4%) |  |
| 31-49 | 84(17.7%) | 284(59.9%) | 106(22.4%) | <0.001 |
| >50 | 3(15.8%) | 11(57.9%) | 5(26.3%) |  |
| **Husband's education level** |  |  |  |  |
| No education | 40(28.2%) | 92(64.8%) | 10(7.0%) |  |
| Primary | 129(34.4%) | 205(54.7%) | 41(10.9%) | <0.001 |
| Secondary | 90(22.6%) | 250(62.8%) | 58(14.6%) |  |
| Higher | 29(13.6%) | 117(54.7%) | 68(31.8%) |  |
| **Husband's occupation** |  |  |  |  |
| Farmer | 61(27.5%) | 140(63.1%) | 21(9.5%) |  |
| Daily Labour | 84(31.5%) | 150(56.2%) | 33(12.4%) |  |
| Employee | 95(23.8%) | 229(57.3%) | 76(19.0%) | <0.001 |
| Businessman | 48(20.0%) | 145(60.4%) | 47(19.6%) |  |
| **Toilet Facility** |  |  |  |  |
| Hygienic | 77(19.0%) | 242(59.6%) | 87(21.4%) | <0.001 |
| Nonhygienic | 211(29.2%) | 422(58.4%) | 90(12.4%) |  |
| **Drinking Water** |  |  |  |  |
| Improved | 235(26.3%) | 518(57.9%) | 142(15.9%) | 0.130 |
| Non improved | 53(22.6%) | 146(62.4%) | 35(15.0%) |  |

Note: †P-values are reported from the chi-square test; the Pearson χ2 statistic is after adjusting the complex survey design using second-order Rao–Scott corrections.
