# Supplementary material for: Machine learning prediction of nutritional status among pregnant women in Bangladesh: Evidence from Bangladesh demographic and health survey 2017–18
Source: PLoS One. 2024 May 31;19(5):e0304389. doi: 10.1371/journal.pone.0304389 (PMC11142495; doi:10.1371/journal.pone.0304389)
Supplement: S2 Table — (DOCX) [file pone.0304389.s002.docx]

**S2 Table:** Background characteristics of the study pregnant women in Bangladesh.

| **Variables** | **Categories** | **Percent (%)** | **Frequency(n=1129)** |
| --- | --- | --- | --- |
| **Body mass index** | Underweight | 25.5 | 288 |
|  | Normal weight | 58.8 | 664 |
|  | Overweight/Obese | 15.7 | 177 |
| **Age** | 15-19 | 27.8 | 314 |
|  | 20-24 | 34.4 | 388 |
|  | 25-29 | 22.6 | 255 |
|  | 30-34 | 12.8 | 144 |
|  | 35-49 | 2.5 | 28 |
| **Division** | Barisal | 10.1 | 114 |
|  | Chittagong | 15.4 | 174 |
|  | Dhaka | 15.3 | 173 |
|  | Khulna | 10.7 | 121 |
|  | Mymensingh | 12.9 | 146 |
|  | Rajshahi | 10.7 | 121 |
|  | Rangpur | 11.2 | 126 |
|  | Sylhet | 13.6 | 154 |
| **Type of place of residence** | Urban | 35.4 | 400 |
|  | Rural | 64.6 | 729 |
| **Highest educational level** | No education | 4.3 | 48 |
|  | Primary | 26.8 | 303 |
|  | Secondary | 48.5 | 548 |
|  | Higher | 20.4 | 230 |
| **Religion** | Muslim | 91.7 | 1035 |
|  | Non-Muslim | 8.3 | 94 |
| **Wealth status** | Poorest | 20.5 | 231 |
|  | Poorer | 20.1 | 227 |
|  | Middle | 18.8 | 212 |
|  | Richer | 20.5 | 232 |
|  | Richest | 20.1 | 227 |
| **Total children ever born** | 0 | 39.3 | 444 |
|  | 1-2 | 49.9 | 563 |
|  | 3-4 | 9.2 | 104 |
|  | ≥ 5 | 1.6 | 18 |
| **Number of living children** | 0 | 41.5 | 469 |
|  | 1-2 | 50.5 | 570 |
|  | ≥3 | 8.0 | 90 |
| **Current pregnancy wanted** | No | 25.0 | 282 |
|  | Yes | 75.0 | 847 |
| **Currently breastfeeding** | No | 94.0 | 1061 |
|  | Yes | 6.0 | 68 |
| **Husband’s education level** | No education | 12.6 | 142 |
|  | Primary | 33.2 | 375 |
|  | Secondary | 35.3 | 398 |
|  | Higher | 19.0 | 214 |
| **Respondent currently working** | No | 67.2 | 759 |
|  | Yes | 32.8 | 370 |
| **Husband/partner's age** | <30 | 56.3 | 636 |
|  | 31-49 | 42.0 | 474 |
|  | >50 | 1.7 | 19 |
| **Access to mass media** | No | 35.3 | 398 |
|  | Yes | 64.7 | 731 |
| **Husband/partner's occupation** | Farmer | 19.7 | 222 |
|  | Daily Labour | 23.6 | 267 |
|  | Employee | 35.4 | 400 |
|  | Businessman | 21.3 | 240 |
| **Toilet facility** | Hygienic | 36.0 | 406 |
|  | Nonhygienic | 64.0 | 723 |
| **Drinking water** | Improved | 79.3 | 895 |
|  | Non improved | 20.7 | 234 |
