# Supplementary material for: Machine learning prediction of nutritional status among pregnant women in Bangladesh: Evidence from Bangladesh demographic and health survey 2017–18
Source: PLoS One. 2024 May 31;19(5):e0304389. doi: 10.1371/journal.pone.0304389 (PMC11142495; doi:10.1371/journal.pone.0304389)
Supplement: S3 Table — (DOCX) [file pone.0304389.s003.docx]

**S3 Table:** Evaluation of prediction performance (%) of different ML Algorithms for the overall nutritional status and underweight and overweight/Obese class **without any FT method.**

| Nutritional  status |  | DT | RF | KNN | NB | LR | SVM | ADB | GB | XGB | Bagging |
| --- | --- | --- | --- | --- | --- | --- | --- | --- | --- | --- | --- |
| Overall | Accuracy | 68.17 | **73.79** | 68.27 | 51.34 | 54.82 | 54.72 | 56.32 | 61.96 | 73.56 | 70.34 |
|  | Kappa | 44.09 | **53.65** | 47.90 | 29.45 | 30.43 | 33.24 | 30.43 | 40.87 | 52.63 | 48.23 |
|  | Precision | 62.38 | **68.76** | 64.05 | 52.15 | 53.07 | 54.84 | 54.51 | 60.01 | 67.92 | 64.97 |
|  | Recall | 62.71 | **69.06** | 65.38 | 53.01 | 53.68 | 55.52 | 55.02 | 60.54 | 68.39 | 65.55 |
|  | f1 score | 62.39 | **68.65** | 63.53 | 51.52 | 53.20 | 54.46 | 54.40 | 60.02 | 67.94 | 65.14 |
| Underweight | Precision | 67.16 | **73.37** | 63.85 | 56.73 | 60.54 | 60.85 | 59.45 | 63.18 | 71.00 | 65.47 |
|  | Recall | 62.21 | **67.28** | 76.50 | 64.06 | 62.21 | 65.90 | 59.45 | 58.53 | 65.44 | 67.28 |
|  | f1 score | 64.59 | **70.19** | 69.60 | 60.17 | 61.36 | 63.27 | 59.45 | 60.77 | 68.11 | 66.36 |
|  | AUC | 58.00 | 63.00 | 53.00 | 64.00 | 55.00 | 54.00 | 68.00 | **73.00** | 70.00 | 69.00 |
| Overweight  /Obese | Precision | 68.27 | 72.56 | 72.30 | 52.17 | 54.37 | 53.78 | 55.66 | 65.44 | **74.53** | 73.74 |
|  | Recall | 78.45 | 86.19 | 85.08 | 66.30 | 61.88 | 66.85 | 67.96 | 78.45 | **87.29** | 80.66 |
|  | f1 score | 73.01 | 78.79 | 78.17 | 58.39 | 57.88 | 59.61 | 61.19 | 71.36 | **80.41** | 77.04 |
|  | AUC | 58.00 | 65.00 | 50.00 | 66.00 | 57.00 | 57.00 | 69.00 | **75.00** | 74.00 | 70.00 |
