# Supplementary material for: Machine learning prediction of nutritional status among pregnant women in Bangladesh: Evidence from Bangladesh demographic and health survey 2017–18
Source: PLoS One. 2024 May 31;19(5):e0304389. doi: 10.1371/journal.pone.0304389 (PMC11142495; doi:10.1371/journal.pone.0304389)
Supplement: S4 Table — (DOCX) [file pone.0304389.s004.docx]

**S4 Table:** Feature importance ranking for the best performing algorithm.

| Features | MDI | PI |
| --- | --- | --- |
| Respondent's age | 0.1415794 | 0.1632107 |
| Region | 0.1577798 | 0.0585284 |
| Place of residence | 0.0517586 | 0.0346154 |
| Highest educational level | 0.0805977 | 0.0451505 |
| Wealth index | 0.1190085 | 0.0580268 |
| Total children ever born | 0.0352419 | 0.0100334 |
| Number of living children | 0.0346621 | 0.0105351 |
| Current pregnancy wanted | 0.0442546 | 0.0130435 |
| Husband's education level | 0.0970137 | 0.0479933 |
| Husband's age | 0.0593484 | 0.0591973 |
| Access to Mass Media | 0.0443494 | 0.0337793 |
| Husband's occupation | 0.0927188 | 0.0510033 |
| Toilet Facility | 0.0416874 | 0.0252508 |
