# Supplementary material for: Machine learning prediction of nutritional status among pregnant women in Bangladesh: Evidence from Bangladesh demographic and health survey 2017–18
Source: PLoS One. 2024 May 31;19(5):e0304389. doi: 10.1371/journal.pone.0304389 (PMC11142495; doi:10.1371/journal.pone.0304389)
Supplement: S1 File — (DOCX) [file pone.0304389.s005.docx]

**Supplement A: Description of ML Algorithms**

**1. Logistic Regression:** Logistic Regression is a statistical model used to predict the probabilities for classification problems with two possible outcomes for a given set of predictors. It employs maximum likelihood to estimate parameters and applies the logistic function to compress the output of a linear equation between 0 and 1 [1]. The model is presented as follows,

$$\log(p(X)/(1-p(X) ))= \boldsymbol{X}\beta$$

Where *p* represents the probability of a particular outcome for an individual.

**2. Decision tree:** A decision tree (DT) is a predictive algorithm that uses a tree-based structure and is supervised in nature. This algorithm represents each factor as an internal tree node, and each leaf node represents a particular class [2]. It is commonly used for various disease predictions, where the output variable is categorical or continuous. The predictors or independent variables can be of any type, such as categorical or continuous data, textual data, or graphs. A classification tree is generated if the output variable is categorical or discrete, whereas a regression tree is generated for continuous output variables.

**3. Random Forest:** Random Forest (RF) is an ensemble learning-based classification method that builds many decision trees during training. The final output combines the outcome class of individual decision trees [3], [4].

**4. K-nearest neighbor:** K-nearest neighbor (KNN) is a robust and versatile classifier that belongs to the supervised learning family of algorithms. It is a non-parametric algorithm, making no explicit assumptions about the data distribution. The algorithm stores all available cases and classifies new cases based on a similarity measure. A majority vote of its neighbors classifies a case, and it is assigned to the most common class amongst its k nearest neighbors measured by a distance function [5], [6].

**5. Support Vector Machine:** A support vector machine (SVM) is a supervised machine learning algorithm that aims to identify a reproducible hyperplane of n dimensions that maximizes the distance between support vectors of two class labels. SVM models work well when there are more variables than samples and when the sample size is small. Although SVM models are memory efficient, they do not provide probability estimates directly and require an expensive five-fold cross-validation process [7], [8].

**6. Naïve Bayes:** Naïve Bayes is a non-artificial neural network that uses Bayes' Theorem as a probability-based algorithm to learn mathematically from data. Bayes' rule, also known as Bayes' Theorem, is a method that determines the probability of an event based on knowledge of conditions that may be related to the event. Naive Bayes is a simple probabilistic model that can handle high-dimensional data with ease [9]. The following equation gives the mathematical representation of NB:

$$P\left( X | C \right)=\prod_{i=1}^{n} P\left( x_{i} | C \right)$$

$$P\left( x_{i} | C \right)=\frac{P\left( x_{i}\cap C \right)}{P\left( C \right)}$$

*X*, $x_{i}$*,* and *n,* indicate the set of all regressors, a single regressor, and the number of regressors. *C* represents the number of the classes or possibilities, 𝑃(𝑋|𝐶) represents the likelihood.

While the probability distribution is not restricted, a normal distribution is often assumed to simplify calculations. The primary rule of this approach is to choose an outcome class with the highest probability, given the features [1].

**7. eXtreme Gradient Boosting:**  eXtreme Gradient Boosting (XGB) is an ensemble machine learning algorithm that uses decision trees and a gradient boosting framework [10]. By combining a series of weak classifiers, boosting produces an averaged classifier that is powerful and effective in reducing variance. It is a valuable technique for solving classification and prediction problems. XGB employs decision trees that are optimized for speed and improved models. [8].

**8. Adaptive Boosting:** Adaptive boosting (ADB) is a method for improving the performance of simple supervised methods. It was developed by Schapire and Freund in 2013 [11]. The foundation of this method is building a weak classifier for each regressor and then evaluating the performance. Any data sample incorrectly classified will be adjusted sequentially to improve performance.

**9. Gradient boosting***:* Gradient boosting is a set of powerful machine learning techniques that have proven successful in many practical applications. These techniques are highly adaptable to the application's specific needs, such as training on different loss functions [12].

**10. Bagging:** Bagging is an ensemble method that generates individuals for its ensemble by training each classifier on a random redistribution of the training set. To create the training set for each classifier, N examples are randomly drawn with replacements from the original training set, where N is the size of the original training set. This may result in many original examples being repeated in the resulting training set, while others may be omitted [13].

**Supplement B: Description of Performance Evaluation Parameter**

**Kappa statistics:** Kappa statistics helps handle multi-class and imbalanced classification issues. It represents the ratio between predicted and actual classifications in a dataset while considering the actual taxonomy. Cohen's Kappa range is ≤1[14].

**Precision:** Precision is also called Positive Predictive Value (PPV) and measures the proportion of true and false positives among the positive results. Mathematically, it is expressed as:

$$Precision=(True Positive)/(True Positive+Flase Positive)\times100$$

**f1 score:** The f1 score (F-Measure) is a composite metric that combines precision and recall into a single metric. It uses the harmonic mean instead of the arithmetic mean to regularize the extreme values more. f1 is expressed mathematically as a harmonic mean.

$$f1 score=(2\times(Precision\times Recall)/(Precision+Recall))\times100$$

**Recall (Sensitivity):** Recall (Sensitivity) is the ratio of the total number of positive samples correctly classified to the sum of the total number of positive and negative samples classified. High recall scores indicate that the classifier has correctly identified many positive class instances and a few false negatives. Recall can be expressed mathematically as:

$Recall=\frac{True Positive}{True Positive+Flase Negative}\times100$

**Accuracy***:* Accuracy refers to the overall correctness of the model and is the proportion of correctly predicted instances for each class to the total number of sample cases for a given algorithm.

$$Accuracy=(Flase Positive+Flase Negative)/(True Positive+True Negative+Flase Positive+Flase Negative)\times100$$

**Area Under the ROC Curve:** Area Under the ROC Curve (AUC) is a measure of the ability of a model to distinguish between cases and non-cases. The AUC of the ROC is calculated based on predicted and actual outcomes and is averaged for the test datasets to compare the discriminating powers of the algorithms. The AUC ranges from 0 to 1, where a perfect classifier has an AUC of 1. The lower bound for random classification is 0.5, and classifiers with an AUC significantly greater than 0.5 have some ability to discriminate between cases and non-cases[15]. The AUC Curve is plotted with TPR on the y-axis and FPR on the x-axis.

***Reference:***

[1] M. M. Khudri, K. K. Rhee, M. S. Hasan, and K. Z. Ahsan, “Predicting nutritional status for women of childbearing age from their economic, health, and demographic features: A supervised machine learning approach,” *PLOS ONE*, vol. 18, no. 5, p. e0277738, May 2023, doi: 10.1371/journal.pone.0277738.

[2] J. R. Quinlan, “Induction of decision trees,” *Machine Learning*, vol. 1, no. 1, pp. 81–106, Mar. 1986, doi: 10.1007/BF00116251.

[3] M. I. Hossain *et al.*, “Performance Evaluation of Machine Learning Algorithm for Classification of Unintended Pregnancy among Married Women in Bangladesh,” *Journal of Healthcare Engineering*, vol. 2022, 2022, doi: 10.1155/2022/1460908.

[4] T. Zhang, J. Su, Z. Xu, Y. Luo, and J. Li, “Sentinel-2 satellite imagery for urban land cover classification by optimized random forest classifier,” *Applied Sciences (Switzerland)*, vol. 11, no. 2, pp. 1–17, 2021, doi: 10.3390/app11020543.

[5] J. R. Khan, S. Chowdhury, H. Islam, and E. Raheem, “Machine Learning Algorithms To Predict The Childhood Anemia In Bangladesh,” *Journal of Data Science*, vol. 17, no. 1, pp. 195–218, Feb. 2021, doi: 10.6339/jds.201901_17(1).0009.

[6] T. M. Cover and P. E. Hart, “Nearest Neighbor Pattern Classification,” *IEEE Transactions on Information Theory*, vol. 13, no. 1, pp. 21–27, 1967, doi: 10.1109/TIT.1967.1053964.

[7] M. Awad and R. Khanna, “Efficient learning machines: Theories, concepts, and applications for engineers and system designers,” *Efficient Learning Machines: Theories, Concepts, and Applications for Engineers and System Designers*, no. July 2018, pp. 1–248, 2015, doi: 10.1007/978-1-4302-5990-9.

[8] O. N. Chilyabanyama *et al.*, “Performance of Machine Learning Classifiers in Classifying Stunting among Under-Five Children in Zambia,” *Children*, vol. 9, no. 7, Jul. 2022, doi: 10.3390/children9071082.

[9] T. M. Cover, “Geometrical and Statistical Properties of Systems of Linear Inequalities with Applications in Pattern Recognition,” *IEEE Transactions on Electronic Computers*, vol. EC-14, no. 3, pp. 326–334, 1965, doi: 10.1109/PGEC.1965.264137.

[10] J. Friedman, T. Hastie, and R. Tibshirani, “Additive Logistic Regression,” *The Annals of Statistics*, vol. 28, no. 2. pp. 337–374, 2000.

[11] R. E. Schapire, *Boosting: Foundations and Algorithms*, vol. 42, no. 1. 2013. doi: 10.1108/03684921311295547.

[12] A. Natekin and A. Knoll, “Gradient boosting machines, a tutorial,” *Frontiers in Neurorobotics*, vol. 7, no. DEC, 2013, doi: 10.3389/fnbot.2013.00021.

[13] R. Maclin, “Popular Ensemble Methods : An Empirical Study Popular Ensemble Methods : An Empirical Study,” *Journal of artificial intelligence research*, vol. 11, no. July, pp. 169–198, 2016.

[14] W. Yu, T. Liu, R. Valdez, M. Gwinn, and M. J. Khoury, “Application of support vector machine modeling for prediction of common diseases: the case of diabetes and pre-diabetes,” *BMC medical informatics and decision making*, vol. 10, no. 1, 2010, doi: 10.1186/1472-6947-10-16.

[15] A. Liaw and M. Wiener, “Classification and Regression by randomForest,” *R News*, vol. 2, no. 3, pp. 18–22, 2002.
